# Differential associations of NFL and GFAP with neuropsychiatric symptoms by amyloid status across the Alzheimer’s disease continuum

**DOI:** 10.3389/fneur.2026.1782816

**Published:** 2026-04-10

**Authors:** Jiaonan Wu, Haitang Jiang, Rui Wang, Xinyi Lv, Fang Tang, Xianliang Chai, Yao Zhu, Zhaozhao Cheng, Yan Wu, Qiqiang Tang

**Affiliations:** 1 Cheeloo College of Medicine, Shandong University, Jinan, China; 2 Department of Neurology, The First Affiliated Hospital of USTC, Division of Life Sciences and Medicine, University of Science and Technology of China, Hefei, Anhui, China

**Keywords:** Alzheimer’s disease, biomarkers, glial fibrillary acidic protein, neurofilament light chain, neuropsychiatric symptoms

## Abstract

**Background:**

Neuropsychiatric symptoms (NPS) are key clinical manifestations across the Alzheimer’s disease (AD) continuum and predict worse clinical outcomes, yet their biological correlates remain incompletely understood. It remains unclear whether biomarkers of neuroaxonal injury and astrocytic activation, namely neurofilament light chain (NFL) and glial fibrillary acidic protein (GFAP), are associated with NPS independently of amyloid-*β* (Aβ) pathology or through downstream structural brain changes.

**Methods:**

We conducted a cross-sectional study of 478 individuals from the First Affiliated Hospital of the University of Science and Technology of China, spanning the cognitive spectrum from cognitively unimpaired (CU) to mild cognitive impairment (MCI), AD dementia, and non-AD dementia. NPS were assessed using the Neuropsychiatric Inventory Questionnaire (NPIQ). We measured core AD biomarkers (Aβ42/40, pTau181, and pTau217) and serum NFL and GFAP using single-molecule array (Simoa) assays. Aβ status was determined by amyloid PET or CSF Aβ42/Aβ40 ratio, and cortical thickness was derived from 3D T1-weighted MRI.

**Results:**

NPS burden was substantially higher in both AD dementia and non-AD dementia than in CU or MCI, highlighting the transdiagnostic nature of NPS in dementia syndromes. Associations between serum biomarkers and NPS differed by Aβ status. In Aβ − individuals, serum NFL was associated with global NPIQ burden and multiple symptom domains, whereas in Aβ + individuals, serum GFAP was associated with global NPIQ burden and several symptom domains. Formal interaction analyses confirmed significant effect modification by Aβ status for serum NFL, but not for serum GFAP. Sensitivity analyses excluding extreme NFL values yielded unchanged results.

**Conclusion:**

These findings support an Aβ-dependent dissociation in biomarker correlates of NPS, with stronger NFL-related associations in Aβ − individuals and stronger GFAP-related associations in Aβ + individuals. Our results suggest that biologically distinct pathways may underlie neuropsychiatric manifestations across the cognitive continuum and support biomarker-informed subtyping of NPS in aging and dementia.

## Introduction

Neuropsychiatric symptoms (NPS), encompassing depression, apathy, agitation, and psychosis, represent a pervasive and clinically significant challenge across the entire spectrum of Alzheimer’s disease (AD), manifesting from the preclinical and prodromal stages to overt dementia (1, 2). These symptoms are not merely secondary reactions to cognitive decline but are increasingly recognized as core features of the underlying neuropathological process, contributing independently to accelerated functional decline, increased caregiver burden, and earlier institutionalization (3). The concept of mild behavioral impairment (MBI), which defines persistent NPS in later life as an early risk marker for dementia, provides a framework for identifying individuals in the pre-cognitive stages of neurodegeneration (4–7).

Despite their prognostic and diagnostic importance, the neurobiological underpinnings of NPS in AD remain inadequately defined (8). Neuroimaging studies have linked specific symptom domains to regional brain alterations—for instance, apathy with anterior cingulate cortex dysfunction and depression with frontal-limbic circuit disruptions (9, 10). Indeed, macroscopic brain structural changes, such as regional cortical thinning, have themselves been associated with the presence and severity of NPS (11, 12). However, these structural and metabolic correlations offer limited insight into the specific upstream molecular drivers. The relationship between core AD proteinopathies, as reflected by fluid biomarkers (13, 14), and NPS is complex and inconsistent. Some evidence suggests that plasma phosphorylated tau (pTau) biomarkers, such as pTau181, are associated with MBI in cognitively unimpaired and mild cognitive impairment (MCI) individuals (15). However, a critical gap exists in understanding how these associations are influenced by the presence of cerebral amyloid-*β* (Aβ) pathology, a key initiating factor in the AD cascade.

Beyond the canonical amyloid and tau pathology, biomarkers capturing other facets of neurodegeneration hold promise for elucidating the mechanisms of NPS. Neurofilament light chain (NFL), a marker of axonal damage, is elevated in a wide range of neurological and even primary psychiatric conditions, suggesting sensitivity to generalized brain injury processes (16, 17). Recent longitudinal data in older adults without dementia indicate that plasma NFL, alongside glial fibrillary acidic protein (GFAP)—a marker of astrocytosis (18–21)—can predict the emergence and worsening of NPS over time, and their combination with clinical symptoms improves the prediction of underlying AD pathology (22). This positions NFL and GFAP as dynamic indicators of active brain pathology relevant to behavioral manifestations. However, it remains unclear whether their association with NPS is primarily driven by co-occurring AD pathology or represents an independent pathway to symptom generation. Furthermore, given that macroscopic brain atrophy (e.g., cortical thinning) is both a known correlate of NPS (11, 12) and a potential consequence of the neurodegeneration reflected by these biomarkers (14, 23, 24), its potential mediating role between biomarkers and NPS has not been established, leaving the pathway from molecular pathology to clinical symptoms incompletely mapped.

To address these knowledge gaps, we comprehensively investigated the relationships between fluid biomarkers and NPS across the cognitive continuum, with particular emphasis on the modifying role of Aβ pathology. First, we characterized the burden and profile of NPS across clinical stages and Aβ-defined subgroups. Second, we examined associations between core AD biomarkers (including CSF Aβ42/40, pTau181, and plasma pTau181 and pTau217) and global neuropsychiatric burden. Third, we evaluated the Aβ-stratified associations of serum NFL and GFAP with global and domain-level NPS, and formally tested biomarker-by-Aβ interaction effects. Finally, we assessed whether cortical thickness mediated the observed associations between serum biomarkers and neuropsychiatric burden.

By integrating fluid biomarkers with neuroimaging and detailed clinical phenotyping across a well-characterized cohort spanning the AD continuum, our findings aim to disentangle the complex etiological basis of NPS. This approach will help clarify whether NPS are direct non-cognitive manifestations of AD pathophysiology or arise from parallel neurobiological processes, ultimately informing the development of more targeted early intervention strategies.

## Methods

### Study participants

This analysis included 478 participants from a prospective cohort study based at the First Affiliated Hospital of the University of Science and Technology of China (25). The participants were Han Chinese adults covering a spectrum of cognitive states—cognitively unimpaired (CU), MCI, and dementia—for longitudinal assessment of neuroimaging and fluid biomarkers to improve early and accurate diagnosis of AD. All participants completed standardized neuropsychological evaluations, including the Mini-Mental State Examination (MMSE) and Clinical Dementia Rating (CDR). An accompanying informant was required for independent assessment of daily functioning. Data on demographics, medical history, and family history were systematically collected. Eligible participants were aged 45 years or older, in generally stable health, and able to complete study procedures such as neuroimaging. Each was accompanied by a reliable partner familiar with their daily functioning. Key exclusions comprised major neurological conditions other than suspected neurodegenerative dementia (e.g., Huntington’s disease, multiple sclerosis, brain tumor, epilepsy), significant uncontrolled systemic diseases, and psychiatric disorders such as major depression, bipolar disorder, or schizophrenia that could confound cognitive assessment. CU participants served as controls. Diagnoses of MCI and AD were made in accordance with the 2011 National Institute on Aging–Alzheimer’s Association (NIA-AA) criteria (26, 27). CU participants had a global CDR score of 0. MCI was defined as a global CDR score of 0.5 with a memory box score ≥0.5. AD dementia required a global CDR score ≥1 and fulfillment of the 2011 National Institute on Aging–Alzheimer’s Association criteria. Individuals with cognitive impairment or dementia not attributable to AD were classified as having non-AD dementia. APOE-ε4 carrier status was assigned to participants carrying one or two ε4 alleles. The study protocol was approved by the ethics committee of the First Affiliated Hospital of USTC (2019KY-26). All participants provided written informed consent prior to inclusion.

### Neuropsychiatric assessment

NPS were evaluated using the Neuropsychiatric Inventory Questionnaire (NPIQ), an informant-based instrument assessing behavioral and psychological symptoms in dementia patients over the preceding month (28). The NPIQ covers 12 domains: delusions, hallucinations, agitation/aggression, depression/dysphoria, anxiety, elation/euphoria, apathy/indifference, disinhibition, irritability/lability, aberrant motor behavior, nighttime behavior, and appetite/eating disorders. Symptom severity was rated based on frequency (scored 1–4: occasionally [<once/week], often [∼once/week], frequently [several times/week], or very frequently [daily]) and severity (scored 1–3: mild, moderate, or severe). The total severity score ranges from 0 to 144, with higher scores indicating more severe symptoms. Caregiver distress was also assessed on a 6-point scale (0–5) for each domain, with total distress scores ranging from 0 to 60. For the current study, two complementary domain grouping approaches were applied. The first, a symptom-based syndromic grouping, classified symptoms into: Psychosis (delusions, hallucinations), Affective (depression, anxiety, elation), Behavioral, Activation/Disinhibition (agitation, disinhibition, irritability), Apathy (apathy/indifference), Vegetative (aberrant motor behavior, sleep, appetite changes). The second, a neuroanatomical grouping, mapped symptoms to brain circuitry: Frontal/Executive (apathy, disinhibition, aberrant motor behavior, agitation), Limbic (depression, anxiety, irritability), Psychosis (delusions, hallucinations), Circadian (sleep, appetite changes), both grouping schemes were used to facilitate dimensional and systems-level analysis of neuropsychiatric profiles.

### Fluid biomarker measurement

CSF and blood samples were processed following standardized protocols. After collection, samples were centrifuged and cells were removed. The resulting CSF and plasma were aliquoted into 200 μL volumes and stored at −80 °C until analysis. Biomarker concentrations were determined using single-molecule array (Simoa) technology on an HD-X analyzer (Quanterix). CSF levels of Aβ40, Aβ42, and pTau181 were measured using commercially available kits (Quanterix, 103,714, 101,195). Plasma analyses included Aβ42, Aβ40, and pTau181 (Quanterix, 103,714, 101,195, 103,520), and pTau217 (Quanterix, 104,570). Serum NFL and GFAP levels were quantified using Simoa assays (Quanterix 103,520). All measurements were performed at the Neurodegenerative Disorder Research Center, University of Science and Technology of China (Hefei, China), strictly following the manufacturer’s protocols.

### Aβ status classifications

Aβ status was determined primarily by 18F-florbetapir (AV-45) PET imaging when available. For participants without valid PET data, Aβ status was classified using the CSF Aβ42/Aβ40 ratio. The cutoff for CSF Aβ42/Aβ40 was 0.0642. Participants with a ratio below this threshold were classified as Aβ-positive (A+) (25).

### Neuroimaging acquisition and processing

Magnetic resonance imaging (MRI) was performed within 1 month of clinical evaluation and biofluid collection using a 3.0 T GE DISCOVER 750w scanner. High-resolution 3D-T1 weighted images were acquired with a 3D-Bravo sequence. Cortical thickness measures were derived using FreeSurfer (v6.0) through the standard recon-all pipeline, focusing on regions vulnerable to Alzheimer’s disease pathology. Intracranial volume was included as a covariate in statistical models. A subset of participants underwent 18F-Florbetapir (AV-45) PET imaging on a Siemens Biograph 16HR scanner following established guidelines (29). Each participant received an intravenous injection of 18F-Florbetapir (259.0 ± 25.9 MBq), with PET images reconstructed using ordered subset expectation maximization. Aβ status (positive/negative) was determined by visual assessment from two experienced nuclear medicine physicians. Among these, 439 individuals had complete MRI, and PET data and were included in predictive modeling.

### Statistical analysis

All statistical analyses were performed using R statistical software (version 4.2.1). Descriptive statistics are presented as mean ± standard deviation for continuous variables and count (percentage) for categorical variables. Group differences in demographic and clinical characteristics were assessed using one-way analysis of variance (ANOVA) for age, education, MMSE, and CDR, whereas analysis of covariance (ANCOVA), adjusted for age, sex, APOE ε4 status, and education, was used for biomarker comparisons. *Post hoc* pairwise comparisons were adjusted using Bonferroni correction. Categorical variables were compared using Pearson’s chi-square tests. Associations between biomarkers and NPIQ scores were examined using multiple linear regression models adjusted for age, sex, APOE ε4 status, and education. Standardized beta coefficients (*β*) were derived using the lm.beta package. To formally test effect modification by Aβ status, additional models included the biomarker term, Aβ status, and a biomarker × Aβ interaction term. Sensitivity analyses were performed for serum NFL by excluding observations above the mean +2 SD, +3 SD, +4 SD, and +5 SD thresholds in the Aβ − subgroup. Mediation analyses were conducted using the mediation package to assess whether cortical thickness mediated the relationship between serum biomarkers and NPIQ scores. Models were stratified by Aβ status and adjusted for age, sex, APOE ε4 status, and education. Indirect effects were tested using nonparametric bootstrap with 5,000 simulations. All tests were two-tailed, and *p* < 0.05 was considered statistically significant. Multiple-comparison correction was applied where indicated; *p* values reported in Table 1 are uncorrected.

**Table 1:** Associations between AD core biomarkers and NPIQ total scores.

Participants	Predictor	*β*	*p*
All	CSF pTau181	0.069	0.271
	CSF Aβ42/40	0.008	0.904
	Plasma pTau217	0.095	0.053
	Plasma pTau181	0.147	**0.013**
	Plasma Aβ42/40	−0.009	0.882
A−	CSF pTau181	0.200	0.129
	CSF Aβ42/40	0.070	0.581
	Plasma pTau217	0.087	0.297
	Plasma pTau181	0.077	0.434
	Plasma Aβ42/40	−0.065	0.512
A+	CSF pTau181	0.019	0.795
	CSF Aβ42/40	0.092	0.194
	Plasma pTau217	0.074	0.243
	Plasma pTau181	0.139	0.058
	Plasma Aβ42/40	0.044	0.551

Multiple linear regression models examining the associations of CSF and plasma biomarkers with NPIQ total score. All models were adjusted for age, sex, APOE ε4 status, and education. Results are presented as standardized beta coefficients (*β*) and uncorrected *p*-values.

## Results

### Demographic and clinical characteristics

The study enrolled 478 participants, including 84 CU individuals, 211 with MCI, 146 with AD dementia, and 37 with non-AD dementia (Table 2). Groups differed significantly in age, APOE-ε4 carrier status, and education level (all *p* < 0.05). AD patients had fewer years of education than CU and MCI participants (*p* < 0.001). Scores on the Mini-Mental State Examination (MMSE) and Clinical Dementia Rating (CDR) worsened progressively across diagnostic groups (all *p* < 0.001). Cerebrospinal fluid (CSF) biomarker analysis revealed that CSF Aβ42/40 was significantly lower in the MCI and AD groups compared to the CU group (*p* < 0.001), while CSF pTau181 was elevated in these groups (*p* < 0.001). Non-AD dementia participants exhibited lower CSF pTau181 levels than those with MCI or AD (*p* < 0.05). Among blood-based biomarkers, plasma pTau217 and pTau181 were higher in MCI and AD groups relative to CU (*p* < 0.001), with AD participants showing further elevations compared to MCI (*p* < 0.001). Serum GFAP was elevated in MCI, AD, and non-AD dementia groups compared to CU (*p* < 0.01), and was higher in AD than in MCI (*p* < 0.001). Serum NFL levels were higher in the AD and non-AD dementia groups than in the CU group (*p* < 0.05), and were also elevated in non-AD dementia relative to MCI (*p* < 0.001).

**Table 2:** Demographic characteristics and fluid biomarker levels of the participants.

Characteristic	Total (*n* = 478)	CU (*n* = 84, 17.6%)	MCI (*n* = 211, 44.1%)	AD (*n* = 146, 30.5%)	Non-AD dementia (*n* = 37, 7.7%)
Age, y	63.28 ± 9.07	58.63 ± 9.06	64.86 ± 8.83^a^	63.37 ± 8.26^a^	64.63 ± 10.49^a^
Female, No. (%)	257 (54%)	47 (56%)	104 (49.5%)	91 (62.3%)	14 (41.2%)^c^
APOE-ε4 carriers, No. (%)	209 (45.5%)	27 (32.5%)	91 (46%)^a^	77 (53.1%)^a^	14 (42.4%)^b,c^
Educational Level, y	9.47 ± 4.22	11.1 ± 4.03	9.96 ± 4.00^a^	7.99 ± 4.34^a^	9 ± 3.50
MMSE	18.07 ± 7.98	27.23 ± 2.47	20.82 ± 4.72^a^	10.4 ± 6.07^a,b^	12.47 ± 5.39^a,b^
CDR	0.72 ± 0.59	0 ± 0	0.5 ± 0.0^a^	1.32 ± 0.52^a,b^	1.26 ± 0.51^a,b^
CSF biomarkers, pg/mL					
CSF Aβ42/Aβ40	0.06 ± 0.02	0.09 ± 0.02	0.06 ± 0.02^a^	0.04 ± 0.01^a,b^	0.09 ± 0.02^b,c^
CSF pTau181	158.47 ± 126.17	37.86 ± 12.69	166.76 ± 140.84^a^	191.88 ± 108.61^a^	64.23 ± 62.96^b,c^
Blood biomarkers, pg/mL					
Plasma Aβ42/Aβ40	0.05 ± 0.01	0.06 ± 0.01	0.05 ± 0.01^a^	0.05 ± 0.01^a^	0.06 ± 0.02
Plasma pTau217	0.69 ± 0.53	0.24 ± 0.2	0.64 ± 0.46^a^	1.01 ± 0.44^a,b^	0.6 ± 0.87^a,c^
Plasma pTau181	4.62 ± 2.71	2.7 ± 1.99	4.41 ± 2.6^a^	6.09 ± 2.32^a,b^	3.84 ± 2.88^a,c^
Serum NFL	31.91 ± 26.95	21.24 ± 35.86	28.93 ± 15.49	37.45 ± 29.44^a,b^	51.36 ± 31.7^a,c^
Serum GFAP	252.92 ± 163.67	112.48 ± 73.35	243.02 ± 142.67^a^	346.91 ± 168.44^a,b^	242.37 ± 162.01^a,c^

Data are presented as mean ± standard deviation for continuous variables and count (percentage) for categorical variables. Continuous variables were compared using one-way ANOVA or ANCOVA as appropriate, and categorical variables were compared using chi-square tests. *Post hoc* pairwise comparisons were Bonferroni-corrected when applicable. Aβ status by diagnostic group: CU (Aβ+ = 3, Aβ− = 81), MCI (Aβ+ = 129, Aβ− = 63), AD dementia (Aβ+ = 135, Aβ− = 0), and non-AD dementia (Aβ+ = 0, Aβ− = 30). Participants with missing Aβ status were excluded from these counts. ^a^*p* < 0.05 vs. CU, ^b^*p* < 0.05 vs. MCI, ^c^*p* < 0.05 vs. AD.

### Neuropsychiatric symptoms across clinical stages and Aβ status

**Figure 1:**
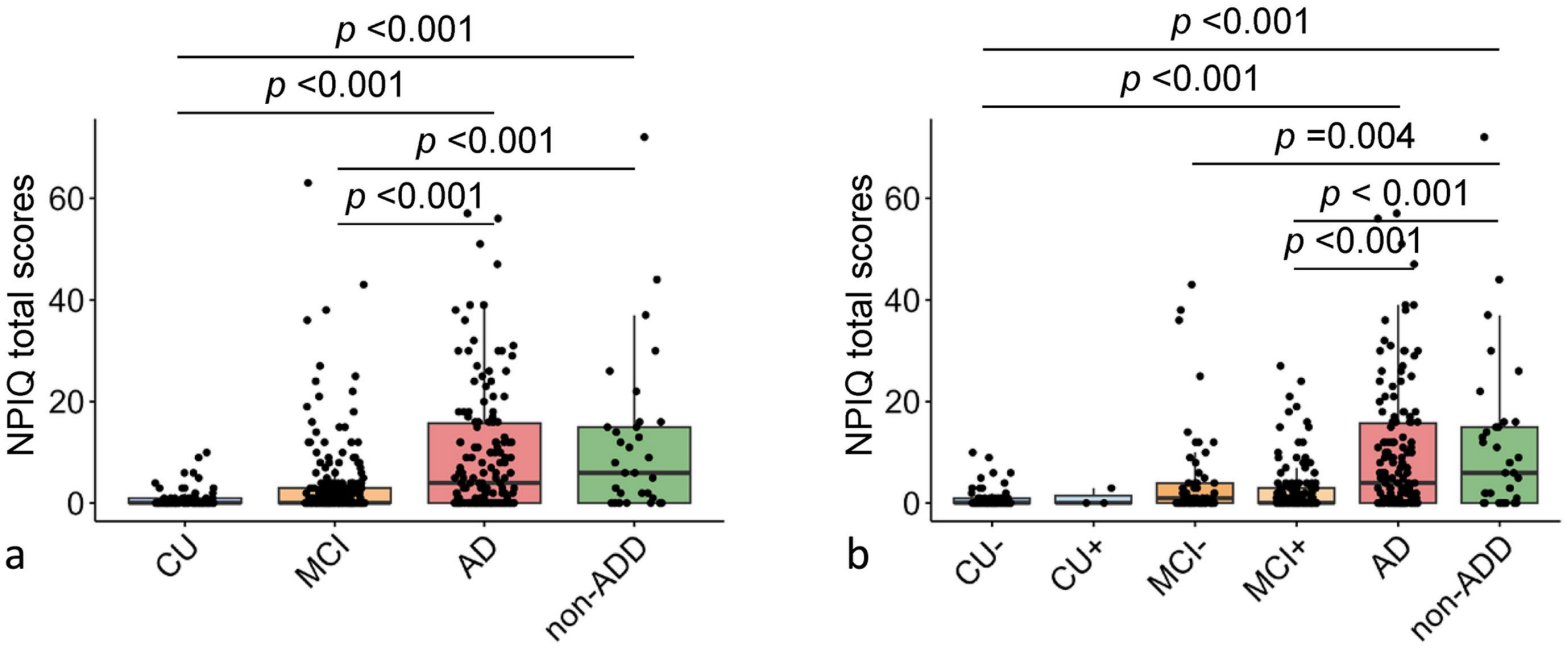
Comparison of neuropsychiatric inventory questionnaire (NPIQ) total scores across groups defined by clinical stage and A*β* status. **(a)** Box plots showing the distribution of NPIQ total scores across clinical diagnostic groups. **(b)** Box plots showing the distribution of NPIQ total scores across groups stratified by both clinical stage and Aβ status. Boxes represent the interquartile range (25th–75th percentiles), the center line indicates the median, and scatter points represent individual participants. Exact *p*-values from pairwise comparisons are shown above brackets, with Bonferroni correction applied for multiple testing. Analyses were adjusted for age, sex, APOE ε4 status, and education. Group definitions: CU −, cognitively unimpaired Aβ-negative; CU +, cognitively unimpaired Aβ-positive; MCI −, mild cognitive impairment Aβ-negative; MCI +, mild cognitive impairment Aβ-positive; AD, Alzheimer’s disease dementia; non-AD dementia, dementia not attributable to AD.

We compared NPIQ total scores across diagnostic groups, with adjustment for age, sex, APOE ε4 status, and education. In the stage-based model (Figure 1a), both CU and MCI groups had significantly lower NPIQ total scores than the AD and non-AD dementia groups (all Bonferroni-adjusted *p* < 0.001), whereas no significant differences were observed between CU and MCI or between AD and non-AD dementia. In the stage- and Aβ-stratified model (Figure 1b), the CU − subgroup had lower NPIQ scores than both the AD and non-AD dementia groups (all *p* < 0.001). Among the MCI subgroups, both MCI − and MCI + participants had lower NPIQ scores than the non-AD dementia group, and MCI + participants also had lower scores than the AD group. No other between-subgroup comparisons reached statistical significance. In a supplementary analysis comparing all Aβ + and Aβ − participants regardless of clinical stage, no significant difference in NPIQ total score was observed.

### Symptom profiles across clinical stages and aβ pathology

**Figure 2:**
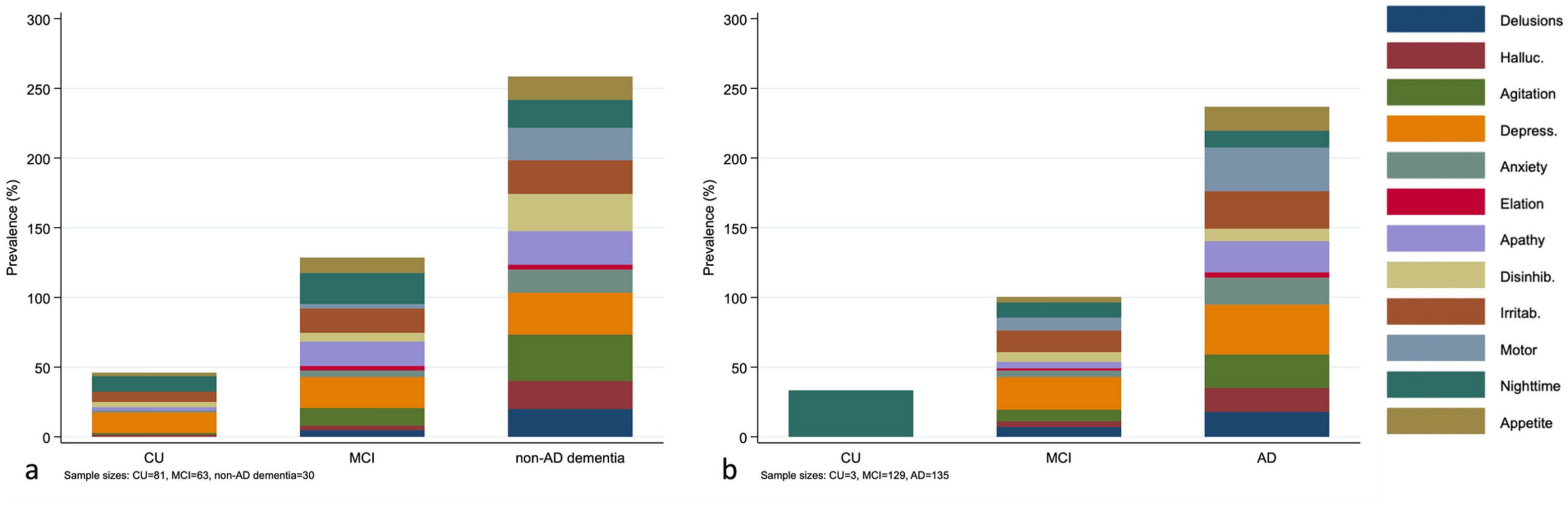
Distribution of NPI subitem prevalence across diagnostic groups within Aβ strata. **(a)** Aβ-negative participants. **(b)** Aβ-positive participants. Bars represent the prevalence of individual NPI subitems within each diagnostic group, including delusions, hallucinations, agitation, depression, anxiety, elation, apathy, disinhibition, irritability, motor behavior, nighttime behavior, and appetite/eating abnormalities. Because these symptom items are not mutually exclusive, stacked values are not intended to sum to 100%. Sample sizes for each subgroup are indicated in the figure. The Aβ-positive CU subgroup was small and should be interpreted cautiously.

The distribution of neuropsychiatric symptom subitems varied across diagnostic groups and according to Aβ status (Figure 2). In the Aβ − panel, the overall burden of NPI subitems was lowest in CU, intermediate in MCI, and highest in non-AD dementia, indicating progressively greater behavioral symptom burden across clinical stages. In the Aβ + panel, the overall burden of NPI subitems was greater in AD dementia than in CU or MCI, with elevations observed across multiple symptom items. This integrated presentation highlights that neuropsychiatric profiles differ more clearly by clinical stage within each A*β* stratum than by Aβ status alone. Because the number of Aβ-positive CU participants was small, symptom frequencies in that subgroup should be interpreted cautiously.

### Associations between Alzheimer’s disease biomarkers and neuropsychiatric symptoms

**Figure 3:**
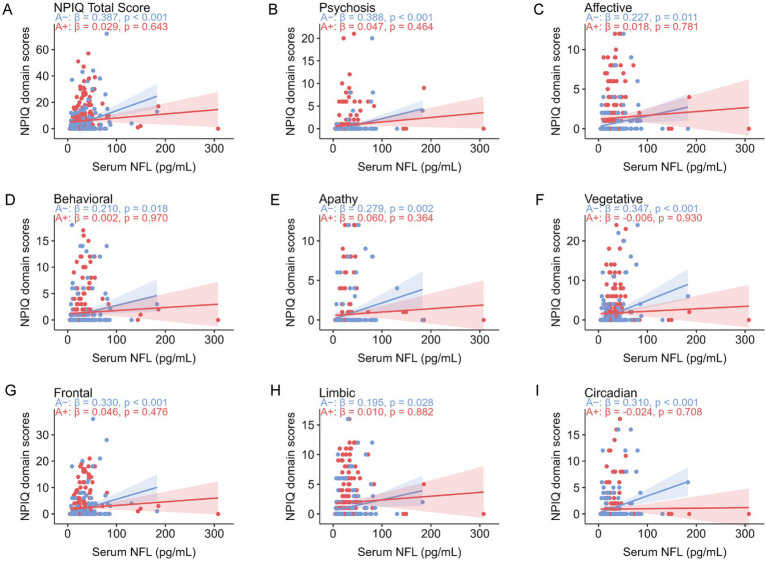
Serum NFL correlations with neuropsychiatric symptoms. Scatter plots display the partial correlations between serum NFL and neuropsychiatric inventory questionnaire (NPIQ) scores, adjusted for age, sex, *APOE* ε4 status, and education levels. **(A)** Correlation with the NPIQ total score. **(B–F)** Correlations with scores from syndromic domains: **(B)** Psychosis, **(C)** Affective, **(D)** Behavioral, **(E)** Apathy, and **(F)** Vegetative symptoms. (**G–I**) Correlations with scores from neuroanatomical domains: **(G)** Frontal, **(H)** Limbic, and **(I)** Circadian. Solid lines represent linear regression fits, with shaded areas indicating the 95% confidence intervals. Standardized beta coefficients (*β*) and *p*-values are shown for each association. Formal biomarker × Aβ interaction analyses are reported in the main text.

**Figure 4:**
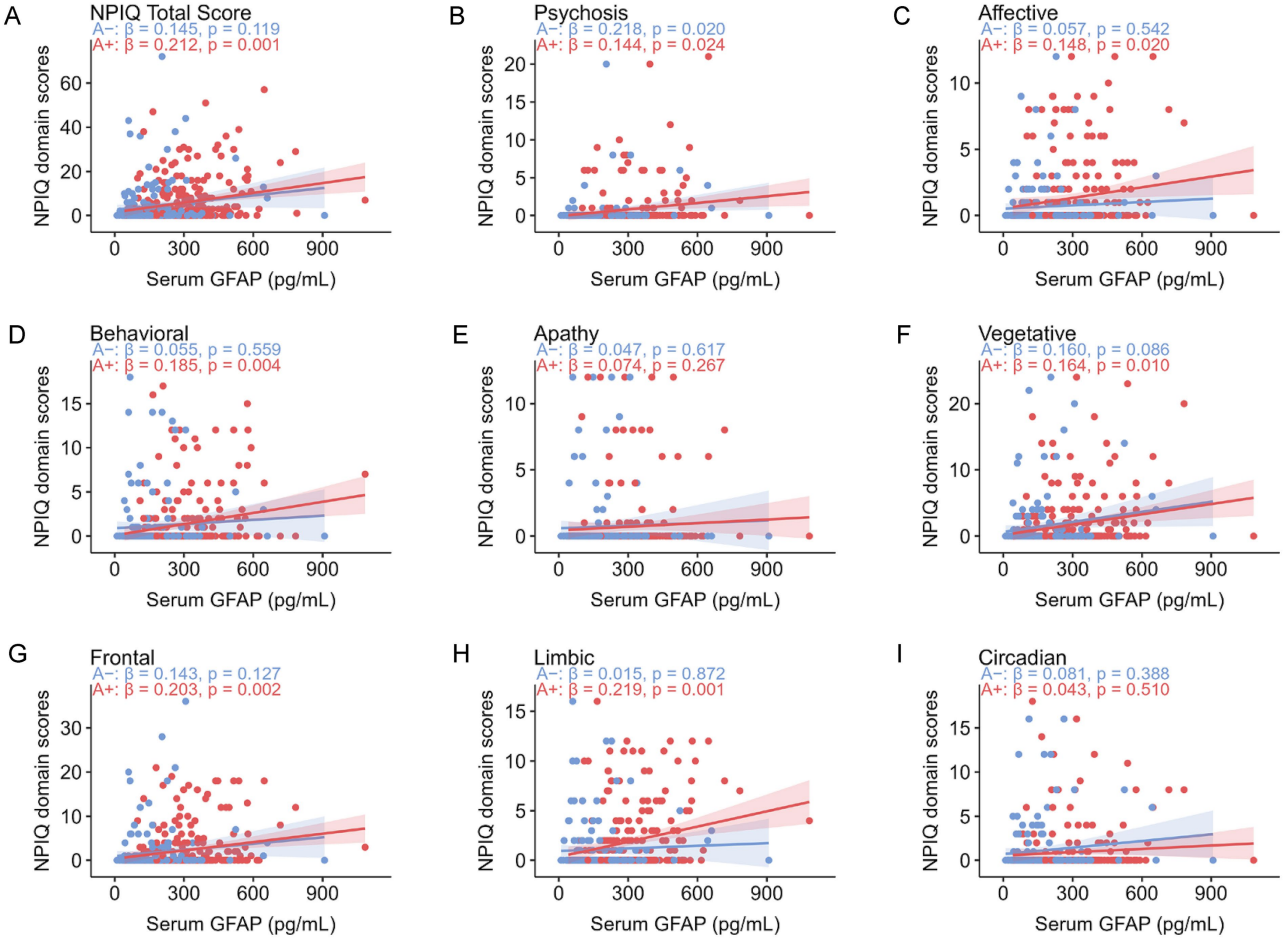
Serum GFAP correlations with neuropsychiatric symptoms. Scatter plots display the partial correlations between serum GFAP and Neuropsychiatric Inventory Questionnaire (NPIQ) scores, adjusted for age, sex, *APOE* ε4 status, and education levels. **(A)** Correlation with the NPIQ total score. **(B–F)** Correlations with scores from syndromic domains: **(B)** Psychosis, **(C)** Affective, **(D)** Behavioral, **(E)** Apathy, and **(F)** Vegetative symptoms. **(G–I)** Correlations with scores from neuroanatomical domains: **(G)** Frontal, **(H)** Limbic, and **(I)** Circadian. Solid lines represent linear regression fits, with shaded areas indicating the 95% confidence intervals. Standardized beta coefficients (*β*) and *p*-values are shown for each association. Formal biomarker × Aβ interaction analyses are reported in the main text.

In models examining core AD biomarkers, plasma pTau181 was the only measure significantly associated with NPIQ total scores in the overall cohort (*β* = 0.147, *p* = 0.013). No other CSF or plasma biomarkers, including Aβ42/40, showed significant associations with global neuropsychiatric burden in the overall, Aβ–, or Aβ + groups (Table 1). Further analyses of serum biomarkers reflecting neuroaxonal injury and astrocytic activation revealed distinct Aβ-dependent associations with NPS (Figures 3, 4). Among Aβ − individuals, serum NFL was positively associated with NPIQ total score and multiple syndromic and neuroanatomical domains. By contrast, serum NFL was not significantly associated with neuropsychiatric measures in the A*β* + subgroup. Serum GFAP showed the opposite pattern: in Aβ + individuals, GFAP was associated with NPIQ total score and several symptom domains, whereas in A*β* − individuals its associations were limited. Formal interaction analyses confirmed significant effect modification by Aβ status for serum NFL in relation to NPIQ total score (interaction *β* = −3.229, SE = 1.093, *p* = 0.003). Significant or nominal NFL × Aβ interactions were also observed for vegetative, frontal, circadian, and psychosis-related symptoms. In contrast, the GFAP × Aβ interaction was not significant for NPIQ total score (interaction *β* = 0.393, SE = 1.290, *p* = 0.761) or for any symptom domain, indicating that the apparent subgroup differences for GFAP are better interpreted as stratified associations rather than statistically confirmed interaction effects. In sensitivity analyses restricted to the Aβ − subgroup, the association between serum NFL and NPIQ total score remained robust after excluding extreme NFL values above the mean +2 SD, +3 SD, +4 SD, and +5 SD thresholds.

### Serum NFL and GFAP associations with neuropsychiatric symptoms are largely direct and not mediated by cortical thickness

**Figure 5:**
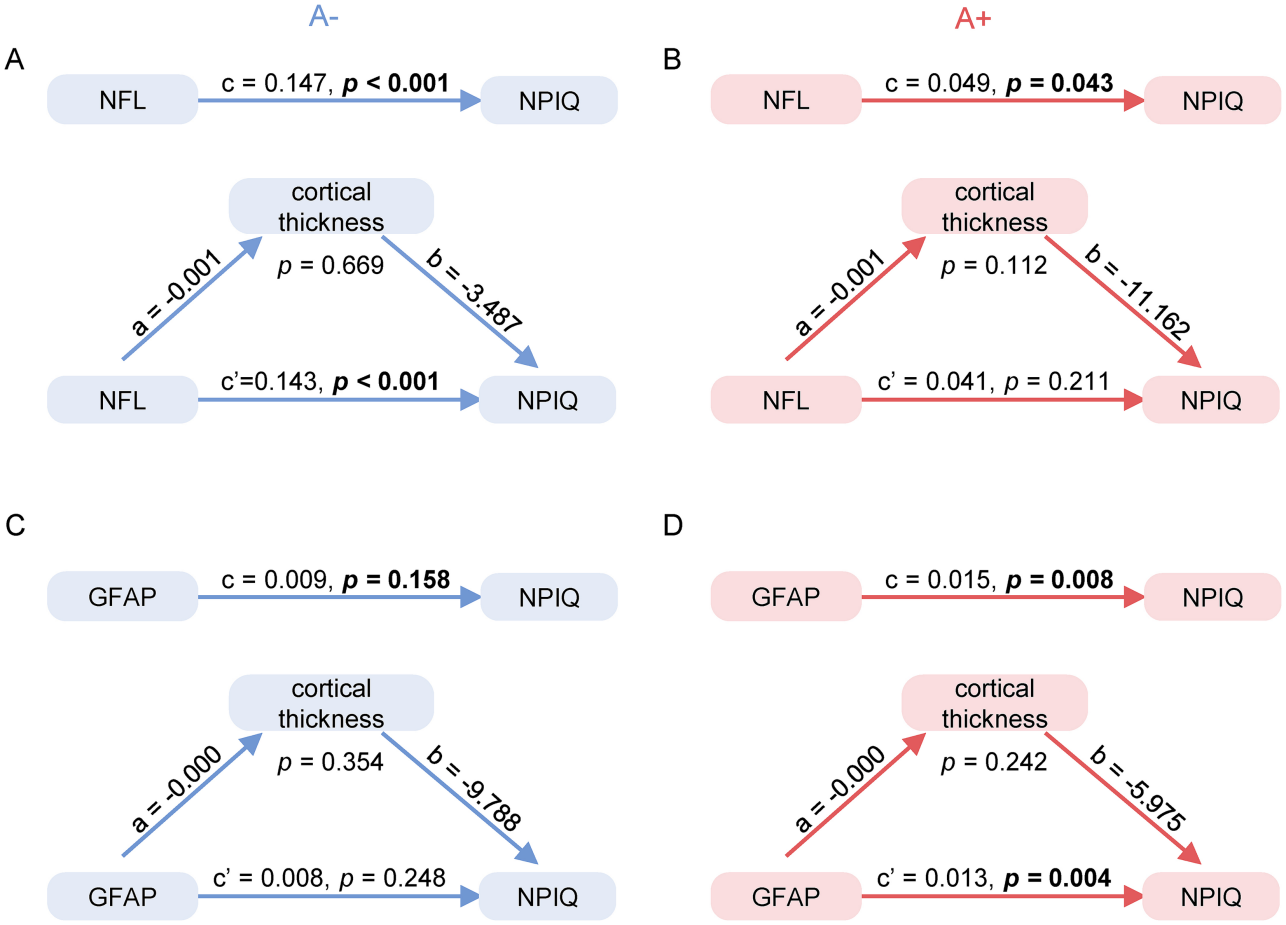
Mediation analysis of serum NFL and GFAP effects on neuropsychiatric symptoms through cortical thickness. Path diagrams illustrate the mediating role of cortical thickness in relationships between serum biomarkers (NFL: **A**,**B**; GFAP: panels **C**,**D**) and neuropsychiatric inventory questionnaire (NPIQ) scores. Path coefficients represent standardized regression coefficients with corresponding *p*-values. For each biomarker, the total effect (c) on NPIQ scores is partitioned into the direct effect (c’) and the indirect effect mediated through cortical thickness (a × b). All effects were estimated using bias-corrected bootstrap confidence intervals based on 5,000 resamples. Analyses were adjusted for age, sex, *APOE* ε4 status, and education levels.

We conducted mediation analyses to assess whether cortical thickness mediated the associations of serum NFL and GFAP with NPIQ scores, stratified by A*β* status (Figure 5). In the Aβ– group (Figure 5A), serum NFL showed a significant total effect on NPIQ scores (*β* = 0.147, *p* < 0.001). Although higher NFL was associated with lower cortical thickness (Path a: *β* = −0.001, *p* = 0.004), cortical thickness did not significantly predict NPIQ scores (Path b: *β* = −3.487, *p* = 0.617). Accordingly, the indirect effect (ACME) was not significant (*p* = 0.669), indicating that the association between NFL and NPS is direct and not mediated by cortical thickness. In the Aβ + group (Figure 5B), the total effect of NFL on NPIQ scores was marginally significant (*β* = 0.049, *p* = 0.043), but neither the direct nor the indirect effect reached significance. For serum GFAP in the Aβ + group (Figure 5D), a significant total effect was observed (*β* = 0.015, *p* < 0.001), driven primarily by a direct effect (*β* = 0.013, *p* = 0.004), with no significant mediation via cortical thickness (ACME *p* = 0.242). In the Aβ– group (Figure 5C), GFAP was not significantly associated with NPIQ scores through either direct or indirect pathways.

## Discussion

This study integrated core AD biomarkers, markers of neuroaxonal injury and astrocytic activation, and detailed neuropsychiatric phenotyping to clarify the biological basis of NPS across the Alzheimer’s disease continuum. Four principal findings emerged. First, the overall burden of NPS was strongly associated with clinical stage, but not with Aβ status alone. Second, NPS burden was similarly high in AD dementia and non-AD dementia, underscoring the transdiagnostic nature of NPS in dementia syndromes. Third, serum NFL and GFAP showed distinct Aβ-dependent associations with neuropsychiatric burden, with stronger NFL-related associations in Aβ − individuals and stronger GFAP-related associations in Aβ + individuals. Fourth, formal interaction analyses supported effect modification by Aβ status for serum NFL but not for serum GFAP, and the NFL findings were robust to sensitivity analyses excluding extreme values.

A pivotal finding was that the overall burden of NPS, as measured by the NPIQ, was comparable between patients with AD dementia and those with non-AD dementia. This indicates that a high NPS burden is a transdiagnostic feature of advanced neurodegenerative states rather than a specific marker of AD pathology. The clinical implication is critical: the presence and severity of NPS in a dementia patient cannot reliably distinguish AD from other causes (28, 30). This underscores the necessity of incorporating disease-specific biomarkers, such as amyloid-PET or CSF pTau, into the diagnostic workup of patients presenting with both cognitive and behavioral complaints to ensure accurate etiological diagnosis and appropriate management.

Furthermore, our data suggest that in pre-dementia stages, the presence of cerebral Aβ pathology alone does not determine the overall severity of NPS. CU and MCI individuals, whether Aβ-positive or Aβ-negative, showed broadly similar global NPIQ burden. This finding suggests that early neuropsychiatric manifestations may reflect heterogeneous processes beyond amyloid deposition alone, including non-AD neurodegenerative mechanisms, psychosocial factors, or other vulnerability states. This observation challenges the notion that NPS in pre-dementia stages are invariably a direct manifestation of emerging AD proteinopathy and calls for a more nuanced, multi-factorial model of their etiology (31, 32).

Against this background, the Aβ-stratified associations of NFL and GFAP become particularly informative. Serum NFL showed strong and broad associations with NPS in Aβ − individuals, especially for global burden, psychosis-related symptoms, vegetative symptoms, and frontal-domain symptoms. Importantly, the formal biomarker-by-Aβ interaction term was significant for NPIQ total score and for several symptom domains, indicating that the NFL–NPS relationship differed meaningfully by Aβ status. These results suggest that, in the absence of canonical AD pathology, neuroaxonal injury may represent an important biological substrate of neuropsychiatric symptoms. This interpretation is consistent with prior work highlighting NFL as a sensitive, albeit non-specific, marker of active brain pathology across psychiatric and neurological disorders (33). In this context, elevated NFL in Aβ − individuals may reflect a final common pathway of neuronal injury arising from vascular, other neurodegenerative, or currently unclassified etiologies that manifest clinically as NPS (34–36).

By contrast, GFAP showed stronger positive associations with global NPS burden and several symptom domains in Aβ + individuals. Given that GFAP reflects astrocytic activation and astrocytes are centrally involved in AD-related neuroinflammatory processes, this pattern is consistent with the possibility that astrocytosis and neuroinflammation are more closely linked to behavioral symptoms within the AD continuum (19, 21, 37). However, the formal GFAP × Aβ interaction term was not significant. Therefore, the GFAP findings should be interpreted as subgroup-specific associations rather than definitive evidence of statistically confirmed effect modification by Aβ status. Taken together, these results suggest that NPS in Aβ − and Aβ + individuals may arise through partially distinct biological pathways, with potential implications for biomarker-based stratification and personalized therapeutic approaches.

Notably, our mediation analyses revealed that the effects of NFL and GFAP on NPS were not transmitted through global cortical thickness. This indicates that the microstructural pathophysiological processes captured by NFL and GFAP—such as synaptic dysfunction (38), axonal damage (39), or neuroinflammation (40)—likely impact neural circuit function and precipitate behavioral symptoms prior to or independently of detectable macroscopic atrophy. This underscores that dynamic, molecular-level pathology may be a more direct correlate of NPS than gross structural imaging metrics.

Among the core AD biomarkers, only plasma pTau181 was significantly associated with global NPS burden in the overall cohort, while measures like CSF Aβ42/40 showed no association. This further supports the notion that tau pathology, being more proximate to neuronal injury (41, 42), may have a more direct link to clinical symptomatology—both cognitive and behavioral—than Aβ pathology alone. The elevation of pTau181 may signify a disease process sufficient to disrupt neural networks and elicit behavioral changes.

Our study has several limitations. First, its cross-sectional design precludes causal inference regarding the temporal relationships between biomarkers and the emergence of NPS. Second, several subgroups were relatively small, particularly the Aβ-positive CU subgroup and the non-AD dementia subgroup, which limits the stability of some stratified symptom-domain estimates. Third, the Aβ − group is likely biologically heterogeneous and may include participants with diverse non-AD pathologies, which may contribute to the broad NFL associations observed in this subgroup. Fourth, although Aβ status was available, harmonized continuous amyloid PET SUVR data were not available across the full analytic sample, precluding uniform SUVR-adjusted sensitivity analyses. Finally, while analyses based on combinations or clustering of NPS may be informative, such analyses would have been exploratory and potentially unstable in the current sample; larger cohorts will be needed to address this question more robustly.

In conclusion, our results demonstrate that NPS are an integral component of the multidimensional clinical presentation of AD, with a biological basis that extends beyond the classical amyloid-tau framework. While NPS lack specificity for AD, their association with serum NFL and GFAP is strongly modulated by the Aβ context. This provides a compelling rationale for using these easily accessible biomarkers to move beyond syndromic classification toward a biological subtyping of neuropsychiatric manifestations in cognitive disorders. Future research should aim to integrate multi-omics data to further delineate the precise pathways from molecular pathology to behavioral expression, thereby paving the way for novel therapeutic interventions targeting the behavioral dimension of AD and other dementias.

## Data Availability

The original contributions presented in the study are included in the article/supplementary material, further inquiries can be directed to the corresponding authors.
